# Nursing in an age of multimorbidity

**DOI:** 10.1186/s12912-018-0321-z

**Published:** 2018-11-29

**Authors:** Siobhan O’Connor, Christi Deaton, Fiona Nolan, Bridget Johnston

**Affiliations:** 1 School of Health and Social Care, Edinburgh Napier University, Edinburgh, UK; 2 Department of Public Health and Primary Care, University of Cambridge, Cambridge, UK; 3 School of Health and Social Care, University of Essex, Colchester, UK; 4 School of Medicine, Dentistry and Nursing, University of Glasgow, Glasgow, UK

**Keywords:** Nurse, Multimorbidity, Chronic illness, Social media, Twitter

## Abstract

**Background:**

A changing sociodemographic landscape has seen rising numbers of people with two or more long-term health conditions. Multimorbidity presents numerous challenges for patients and families and those who work in healthcare services. Therefore, the nursing profession needs to understand the issues involved in supporting people with multiple chronic conditions and how to prepare the future workforce to care for them.

**Methods:**

A descriptive, exploratory study was used to examine the future of nursing in an age of multimorbidity. An hour-long Twitter chat was organised and run by the Florence Nightingale Foundation Chairs of Clinical Nursing Practice Research to discuss this important area of practice and identify what needs to be done to adequately upskill and prepare the nursing profession to care for individuals with more than one long-term illness. Questions were formulated in advance to provide some structure to the online discussion. Data were collected and analysed from the social media platform using NVivo and an analytics tool called Keyhole. Descriptive statistics were used to describe participants and thematic analysis aided the identification of key themes.

**Results:**

Twenty-four people, from a range of nursing backgrounds and organisations, took part in the social media discussion. Five themes encompassing coping with treatment burden, delivering holistic care, developing an evidence base, stimulating learning and redesigning health services were seen as key to ensuring nurses could care for people with multimorbidity and prevent others from developing chronic health conditions.

**Conclusions:**

Multimorbidity is a pressing health issue in today’s society. Changes in nursing research, education and practice are required to help the profession work collaboratively with patients, families and multidisciplinary teams to better manage and prevent chronic illness now and in the future.

## Background

The last century has seen sweeping social changes such as advances in mechanisation and automation that have transformed agriculture, manufacturing, healthcare and other industries. Along with mass urbanisation, where populations of people move from rural areas to towns and cities, and the evolution of information technology these changes have led to a seismic shift in the behaviours and lifestyles of many people [1]. A sedentary way of life is becoming more common which can include an unhealthy diet and a lack of exercise. Harmful habits such as smoking, binge drinking and recreational drug use are also present in contemporary society.

Conversely, a focus on public health and improvements in water and sanitation, housing, transport and education among others have enabled people to live longer, with growing numbers of older adults in many regions of the globe [2]. Older people often have additional complexities in terms of their healthcare needs as long-term conditions such as arthritis, heart disease, Alzheimer’s and dementia tend to become more prevalent with age [3]. As a result, many countries have seen a rapid increase in the numbers of people with one or more long-term conditions such as asthma, diabetes, Chronic Obstructive Pulmonary Disease (COPD), cancer and chronic kidney disease to name a few [4].

These changes have led to people developing multimorbidity, which is the presence of two or more Long-term Conditions (LTCs) occurring at the same time [5]. While some young people are affected, those over the age of 65 make up the majority of individuals with multimorbidity, with social deprivation being a contributing factor [6]. A significant burden of disease exists for those that have multiple LTCs as they complicate treatment and management. For example, polypharmacy (multiple drug use) is associated with adverse drug events [7], a higher risk of falls in the elderly [8] and greater mortality and complications when hospitalised [9]. In addition, multimorbid patients tend to have a poorer quality of life due to the myriad symptoms they need to manage, which can affect their ability to work and enjoy hobbies and personal relationships with family and friends [10, 11]. Informal caregivers who look after those with LTCs can also feel overwhelmed and experience high levels of stress and fatigue [12].

The numbers of patients with multiple co-occurring chronic illnesses is increasing and predictions were that 2.9 million people would have multiple LTCs in England by 2018 [13]. This is expected to add a significant burden to the provision of health services as those with LTCs currently account for 50% of all appointments with family physicians, 65% of all outpatient appointments and 70% of all inpatient day hospital beds in the National Health Service (NHS) in England, accounting for 70% of the costs of healthcare [14]. Similar trends can be seen in the United States with 77% of all adult non-maternal hospitalisations having two or more conditions, making up the vast majority of inpatient costs [15]. People with comorbidities also comprise the bulk of readmissions which are associated with higher healthcare expenditure [16].

For nurses, multimorbidity presents numerous challenges in both hospital and community settings. The continuity and coordination of care can be problematic when nursing people with LTCs. For example, errors can be made when documenting clinical records, communicating with multidisciplinary teams and discharging patients’ home [17, 18] among others. In addition, little clinical research or published guidelines, outside of those provided by the National Institute for Health and Care Excellence [19], exist in relation to comorbidities that can inform nursing education and practice [20]. Nurse managers can also find it difficult to allocate adequate staff patient ratios, with appropriate skills mix, which could compromise the quality of care [21]. Furthermore, a study conducted in primary care revealed nurses struggled to help patients manage numerous medical and social problems, particularly in poorer communities where resources were limited. Nurses needed to negotiate more time to support various self-management practices without being overwhelmed by the complexity of care [22].

As the future of nursing will inevitably include supporting more patients with multiple LTCs in a variety of settings, it is vital that the profession prepares for this. The Florence Nightingale Foundation (FNF), a charitable organisation in the United Kingdom (UK), was set up to further education and professional development of nurses and midwives by enabling them to develop knowledge and skills that bring about innovative research and practice [23]. In an effort to promote this important subject and to discuss the future of nursing in an age of comorbidities, the Florence Nightingale Foundation Chairs of Clinical Nursing Practice Research organised an online discussion via Twitter to engage nurses on the topic and debate what needs to be done to address this area of practice. Social media has the potential to reach a wide audience and has been used to garner nurse’s opinions on health policy [24], educate them on a range of topics [25] and communicate information to patients [26] among others. Therefore, the aim of this study was to examine the views of those who participated in the Twitter chat to understand the challenges and opportunities faced by nurses when caring for patients with multimorbidity.

## Methods

A descriptive, exploratory design was adopted which used an interpretivist approach to examine perspectives on nursing patients with multimorbidity.

### Sample and setting

**Fig. 1:**
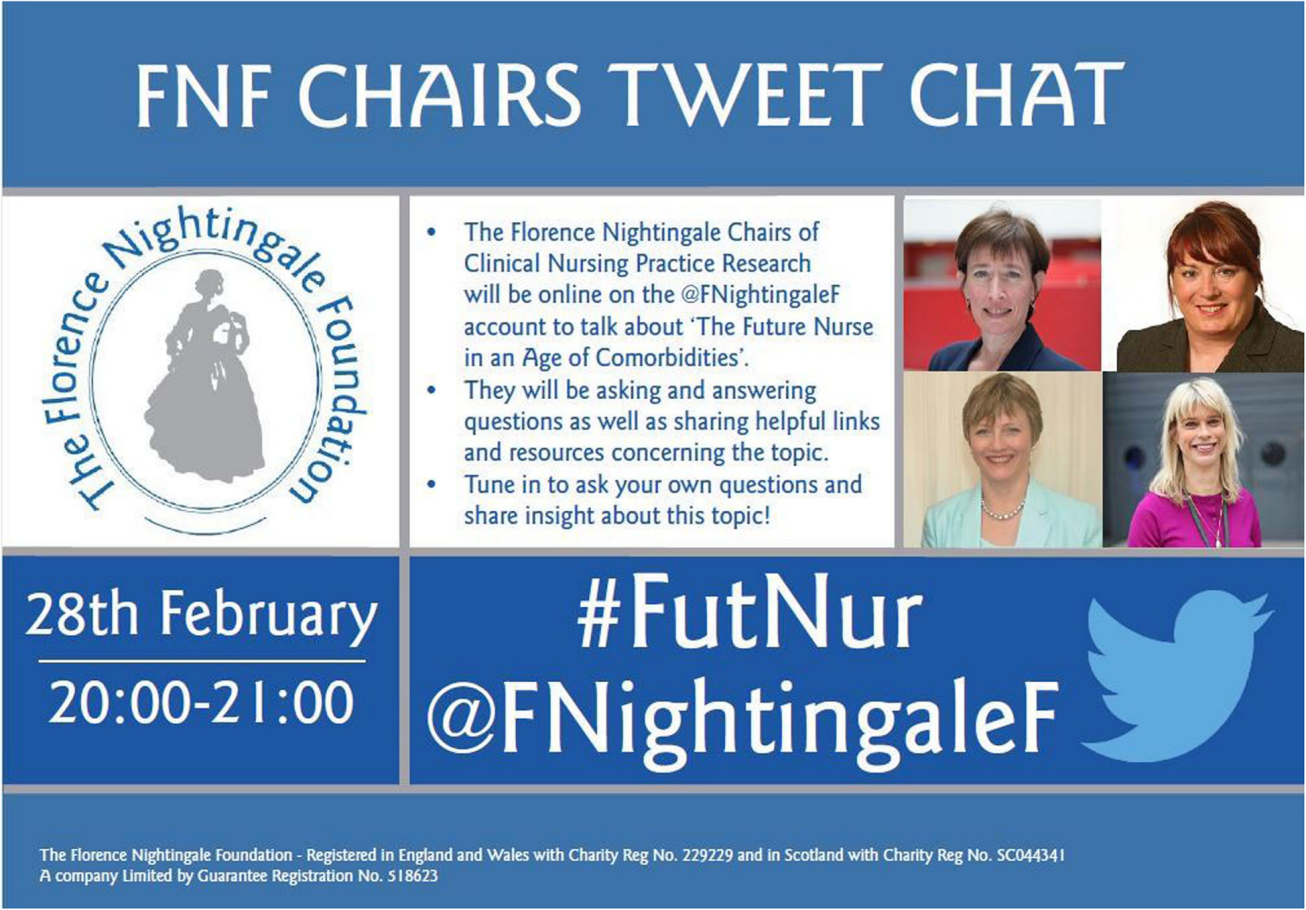
Recruitment flyer for the #FutNur Twitter chat (reproduced with the permission of the author - The Florence Nightingale Foundation)

The Florence Nightingale Foundation advertised the Twitter chat on various social media platforms (see Fig. 1) and sent invitations to some high-profile nurses’ active on these online environments to further promote the event. This helped inform individuals and organisations of the objectives of the virtual focus group taking place on Twitter and how people could get involved. The questions that were posed were published online in advance so people taking part could prepare responses to the topic. The questions were:

1. What are the key issues when caring for people with comorbid conditions?
2. What is the key role for nurses in coordinating care for people with comorbid conditions?
3. Do we need disease specific specialist roles in future or really good and skilled generalists?
4. How will nurses have to change their practice to meet the growing complexities regarding comorbid illness for the future?
5. What are the key skills, training, education needed for the nurses of the future caring for comorbid conditions?

Twitter was chosen as the social media tool as it is an open, public platform, so that anyone with access to a computer or mobile device and the Internet could take part in or observe the chat. Although the 140-character limit can be restrictive, it was felt this feature would encourage participants to communicate succinctly and enable tweets to be read quickly so that conversations throughout the Twitter chat could be followed more easily. A hashtag, which is a searchable term preceded by the number sign, was created to help participants find and keep track of the online discussion as it was happening in real-time. An hour-long tweet chat, using the #FutNur hashtag, ran on Tuesday 28th February 2017 from 20:00 to 21:00. The five questions were posed by the FNF Chairs during the Twitter chat to encourage dialogue on the subject of nursing in an age of multimorbidity.

### Data Collection & Analysis

Ethical approval for secondary analysis of data generated via the Twitter chat was granted by a university ethics committee. Data from the online focus group were collected using the NCapture application on NVivo, which extracted tweets posted under the #FutNur hashtag. An analytics platform called Keyhole (https://keyhole.co//) was also used to gather quantitative data generated by the online discussion and the participants who took part. Thematic analysis was employed to code and categorise the qualitative data [27]. Information posted in tweets which included comments, hashtags and website links were examined for each question posed to generate initial codes that were iteratively refined and developed into overarching themes. This aided an exploration of participants’ views on nursing patients with multimorbidity. A number of analytics tools on the Keyhole platform were used to generate descriptive statistics on the people that took part and the number and reach of tweets and impressions that were generated by the Twitter chat.

### Rigour

To ensure the qualitative analysis was as rigorous as possible a number of approaches were used to enhance trustworthiness in the study results [28]. Robust methods of data collection and analysis were used and a debriefing session with a peer to explore researcher bias helped enhance credibility. A coding clinic between the lead author and a research colleague took place during which samples of data analysis were checked to improve dependability. The rich descriptions provided by participants about how nurses can prepare for and support patients with multimorbidity improved the transferability of findings to other contexts. The triangulation of data from different sources (types of participants on Twitter) helped increase confirmability in the results [29].

## Results

**Fig. 2:**
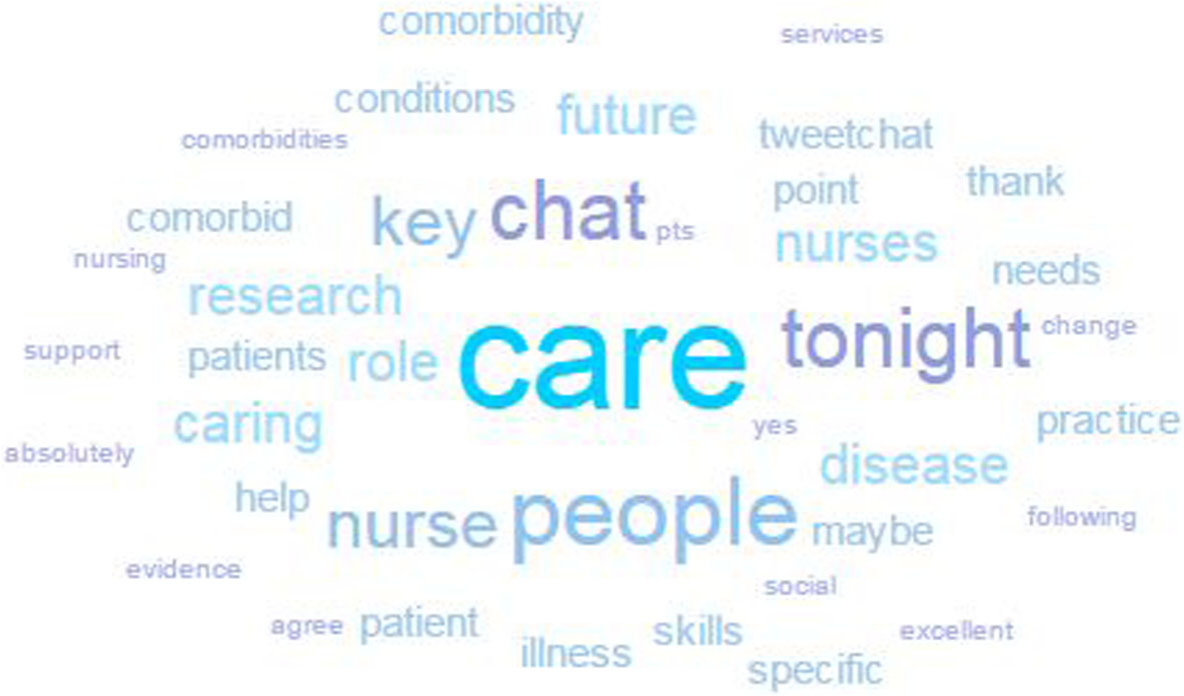
Most frequently used words during the #FutNur Twitter chat

Twenty-four people took part in the online discussion, generating four hundred and seventy six posts to the #FutNur hashtag during the hour-long Twitter chat. The number of tweets per user ranged from 1 to 143, with most participants posting on average 20 tweets over the hour. The most prolific tweeter was the Florence Nightingale Foundation who facilitated the conversation. Some of the FNF Chairs of Clinical Nursing Practice Research also took part using their own Twitter accounts. The #FutNur hashtag reached 54,322 users, the number of people who may have seen the tweets including the followers of those participating in the online chat. In addition, it generated 363,798 impressions on social media, the number of times the users reached may have seen the tweets in their timeline or search results. Based on individual account profiles the characteristics of participants can be seen in Table 1, although some people did not report personal information in their Twitter handle or account profile. The most commonly used words during the #FutNur chat are outlined in the word cloud in Fig. 2.

**Table 1:** Participant Characteristics

Gender	Location	Occupation
Male = 2 Not-specified = 4 Female = 18	Ireland = 1 United Kingdom (non-specific) = 2 Not-specified = 2 Scotland = 2 England = 17	Health Visitor = 1 Nurse Consultant = 1 Clinical Academic Nurse = 1 Staff Nurse = 2 Nurse Lecturer = 2 Nurse Researcher = 3 Nursing Organisation/Group = 4 Nursing Professor = 5 (3 were FNF Chairs) Nursing Leader (Director/Chief Executive) = 5

The questions posed online generated a lot of discussion on what nursing needs to focus on in an age of multimorbidity. These are represented in five overarching themes relating to the questions asked: 1) Coping with Treatment Burden, 2) Delivering Holistic Care, 3) Developing an Evidence Base, 4) Stimulating Learning, and 5) Redesigning Health Services.

### Coping with Treatment Burden

The initial focus of the Twitter chat was the burden patients had to deal with when managing multiple chronic conditions. It was acknowledged that the majority of people seen in the health service in the UK had two or more long-term illnesses, especially those aged over 65. This posed significant challenges for them in terms of scheduling and attending multiple medical appointments with different specialists and managing medication and complicated treatment regimes, which added to the frustration of already being unwell. The extra burden this placed on families and carers was also noted, as their contribution was seen to be important in supporting the person with multimorbidity to manage their complex care needs. One participant highlighted that children can also experience multiple chronic conditions, which needs to be taken into consideration when planning paediatric nursing care (see Table 2: T1Q1, T1Q2, T1Q3, T1Q4).

**Table 2:** Participant quotes related to themes

Participant Quote (Q)	
Theme (T) 1: Coping with Treatment Burden	
T1Q1: “Competing demands, multiple specialists, burden of treatment come to mind for me” - Participant 7 (Nursing Organisation, England)	
T1Q2: “Polypharmacy can become an issue in comorbidity too, confusing for patients and potentially difficult 2 manage” - Participant 16 (Female, Nurse Researcher, England)	
T1Q3: “Also need to consider the burden on family and carers: they can get burnt out & stressed” - Participant 18 (Female, Nursing Leader - Director, England)	
T1Q4: “need to remember it isn’t just the elderly who have co-morbidities Lots kids complex needs” – Participant 19 (Female, Nurse Researcher, Location not specified)	
Theme 2: Delivering Holistic Care	
T2Q1: “Confirming with the patient what those co-morbidities are, ensuring we have the right info & listening” - Participant 6 (Female, Nurse Researcher, England)	
T2Q2: “one thing understanding what matters to them as individuals - not just clinical outcomes” - Participant 2 (Female, Nursing Leader – Chief Nurse, England)	
T2Q3: “Excellent nurse led telephone triage to support a one stop shop works well with our specialist national services” - Participant 5 (Female, Nursing Professor, England)	
T2Q4: “the ability to think and plan care holistically, with critical thinking & analysis underpinning pt. centred care?” - Participant 17 (Female, Nurse Lecturer, England)	
T2Q5: “Caring for the whole person when services, specialties and pathways are fragmented” - Participant 11 (Male, Clinical Academic Nurse, England)	
T2Q6: “absolutely but role of nurse pivotal in bringing it all together and providing continuity of care” - Participant 11 (Male, Clinical Academic Nurse, England)	
Theme 3: Developing an Evidence Base	
T3Q1: “Challenge is multi comorbidities. Good evidence re trajectories for individual diseases” - Participant 9 (Female, Nurse Researcher, England)	
T3Q2: “understanding evidence relating to integrated care models important as is providing care along pathways vs just disease specific” - Participant 10 (Female, Nursing Leader – Director, England)	
T3Q3: “we r working on research that may help to bring 2gether knowledge & 2 understand Overlap btwn conditions of MUS” - Participant 16 (Female, Nurse Researcher, England)	
Theme 4: Stimulating Learning	
T4Q1: “How to balance need for in-depth specialist knowledge with broad ability to coordinate & manage multi-morbidity?” - Participant 7 (Nursing Organisation, England)	
T4Q2: “Nurses will need to be innovators, facilitators, advocates, experts- so will need diverse skill set to meet diverse needs” - Participant 11 (Male, Clinical Academic Nurse, England)	
T4Q3: “we will have Gen. Z’s caring for Gen. Y’s our skills & preparation will need to keep pace with expectations & tech” - Participant 20 (Male, Nursing Professor, England)	
Theme 5: Redesigning Health Services	
T5Q1: “lots nurses can do but need system change to reflect complexity - away from single disease care esp. for older people” - Participant 3 (Female, Staff Nurse, England)	
T5Q2: “How can #FutNur use tech to get closer to where patients are? Rather than expect them to come to us. Best for CYP & elderly” - Participant 19 (Female, Nurse Researcher, Location not specified)	
T5Q3: “Most appro MDT Member Will differ case by case, same as CAF in child safeguarding. Planned meetings, keep on top of needs, reg reassess” - Participant 17 (Female, Nurse Lecturer, England)	
T5Q4: “co-location of social care/support alongside NHS is vital for future joined up care for complex cases” - Participant 1 (Female, Nurse Consultant, England)	

### Delivering Holistic Care

The conversation then moved on to the type of care that nurses need to deliver to support people with multimorbidity. This started by identifying how assessments should be carried out to pinpoint problems stemming from the existence of several chronic conditions occurring together. Some participants felt that comprehensive assessment needs to involve listening to the patients’ story and ensuring their own personal goals are incorporated alongside clinical ones. One individual mentioned a telephone triage service that was being used nationally as a focal point for initial assessments that could be undertaken quickly to establish patients’ needs (see Table 2: T2Q1, T2Q2, T2Q3).

Being person-centred was also seen by some as being critical to looking after people with multiple chronic illnesses, with an emphasis on including all aspects of their health from the physical, to emotional and social wellbeing as a way to address their complex needs. One participant felt this was extremely important given the lack of continuity between some healthcare services, as clinical pathways are not always joined up and cohesive when patients move from primary to secondary care and back. The role of the nurse was thought of as key in this space to ensure coordination of care between multidisciplinary teams for people with multimorbidity (see Table 2: T2Q4, T2Q5, T2Q6).

### Developing an Evidence Base

The lack of evidence on how to care for and adequately support a person with more than one chronic illness was another hot topic of discussion during the Twitter chat. Several participants felt that research was key to understanding the trajectory of multimorbid patients and more needs to be done in this area of nursing as innovative practices are limited. Some shared website links to relevant research and government reports on multimorbidity via Twitter, which outlined statistics on the topic and suggested frameworks that need to be developed to improve the management of patients with multiple long-term conditions. One participant brought up the importance of exploring and creating integrated care models, if future services move towards a more integrated health and social care landscape (see Table 2: T3Q1, T3Q2, T3Q3).

### Stimulating Learning

The type of education that is necessary to enable nurses at all levels to support people with multiple chronic conditions was the fourth major theme that emerged from the Twitter chat. One aspect that was debated, as outlined by the question posed on this issue, was whether it was more important to focus on generalist skills or whether nurses should specialise in specific diseases to address the challenges of multimorbidity. Non-clinical knowledge and skills, in particular the ability to communicate well with multiple professionals, patients and their families, were seen as critical to ensuring care was coordinated and effective. One participant suggested increased education for nurses around technology to ensure they could leverage it in a variety of ways to care for those with complex needs, while another brought up genomics as an area that nurses may need to be trained in in the future to deal with rarer diseases (see Table 2: T4Q1, T4Q2, T4Q3).

### Redesigning Health Services

The final theme of the online discussion centred around the frustration participants felt at the way health services were currently designed. Existing systems, processes and people were not adapting to this new reality, despite the obvious presence of an increasing number of people living with multimorbidity and the challenges it creates for health professionals and patients. Some thought the current focus on single diseases was unhelpful as it led to systems and processes that are unfit for purpose. One suggestion put forward was to make better use of a range of technologies from robotics, to social media and wearable devices, to reach patients at home and give them better access to health services rather than expect them to come to hospital. Other participants believed multidisciplinary team meetings (MDTs) could be used more effectively to bridge the gap between acute and community care, especially for older adults with co-existing chronic illnesses. Holding MDTs in patient’s homes, adding social care professionals into the mix and undertaking regular reassessments were some of the suggestions made in relation to this (see Table 2: T5Q1, T5Q2, T5Q3, T5Q4).

## Discussion

Overall the views of participants involved in the social media discussion were that multimorbidity poses several challenges for patients and their families, which will shape the future of nursing, healthcare and society. The concept of treatment burden experienced by those with chronic conditions was brought up and the myriad of problems faced when living with multiple illnesses day-to-day [30]. Models of treatment burden exist such as those developed by Eton et al. [31], who created a conceptual framework detailing three main domains encompassing self-care, strategies to facilitate self-care and factors that exacerbate burden, which has several subdomains. Sav et al. [32] also identified four components of treatment burden i.e. financial, time and travel, medication and healthcare access. Work has also been done investigating the aspects that help reduce the burden of treatment in people with chronic conditions [33]. These highlight what patients and carers cope with on a regular basis and what might help to address treatment burden. These models could be further developed through research and turned into useful assessment tools to help nurses identify both adult and younger patients who are overwhelmed by the burden of coping with multiple conditions. This could enable care to be better tailored to individual need. Tools to evaluate caregiver burden do exist but many focus on single diseases [34, 35] and a more comprehensive approach is necessary to support family members caring for those with multimorbidity.

From the views expressed by participants, it became clear that some felt current assessment tools in nursing are limited, which means physical, emotional or social problems associated with having several chronic conditions could be missed. For example, Roper’s Activities of Daily Living [36] and Orem’s Self-Care model [37] are frameworks that underpin generic nursing assessments. However, it is argued that these are too rigid and do not go far enough in identifying the holistic needs of individuals, which can lead to inadequate care planning and delivery [38]. Although more comprehensive assessment scales have been developed [39, 40] there are limitations in their design and use [41] and none have been created with multimorbidity specifically in mind. The UK Department of Health’s framework on comorbidities [42] outlines seven system-wide actions that need to be implemented, one of which advocates for joint guidelines and algorithms for preventing and managing multiple long-term illnesses. A strong evidence base was called for from participants in the Twitter chat and so further research on how to construct more inclusive assessments and develop guidelines that incorporate the complexities of having multiple chronic conditions is needed. This future work could be co-designed with people living with multimorbidity to support holistic nursing care and aid the transition of comorbid patients between services.

The need to better educate nurses who care for people with multimorbidity was another finding that emerged from the Twitter chat. Foundational and Continuing Professional Development (CPD) training is necessary to teach nurses about the intricacies of supporting people with several chronic diseases. While the topic is often incorporated in pre-registration nursing education, a disease specific approach is often taken in higher education [43]. In addition, postgraduate qualifications and CPD training tend to adopt this perspective as career progression in advanced practice centres on disease specialities. While specialist knowledge and skills will be needed a range of generic expertise should be incorporated into nursing education to help address the needs of comorbid patients. This could include how to use technology to support patient self-management practices at home and coordinate care between multidisciplinary teams [44, 45]. With the advent of precision medicine, which advocates for healthcare interventions to be tailored to the needs of individuals based on their biological make-up [46], specific topics such as polypharmacy and genomics may be needed in future nursing curricula. Big Data may be another useful avenue to explore as a range of diverse datasets could assist in understanding the complexities of multimorbidity [47]. This would enable nurses to participate in cutting edge research that includes cohorts of people with multiple chronic conditions and to prepare for changes in future healthcare environments [46].

A final point made by participants in the Twitter chat concentrated on changes that may be necessary at the health systems level to enable a more person-centred approach to managing multimorbidity and ideally preventing its occurrence. The siloed approach to treating diseases in isolation was considered by some in the online discussion as a key area to address. MDTs in combination with case management have been tried and tested as one means of providing a more integrated approach to caring for patients with multiple chronic conditions [48]. A consideration that could be beneficial, not discussed during the chat, is hospital care that is coordinated by generalists rather than dictated by specialist disease specific clinicians [49]. The technologies noted during the chat, such as wearable devices and robotics, along with others such as social media and mobile apps are being developed, evaluated and implemented to encourage healthy lifestyles and behaviours [50, 51]. This could be one way to promote preventative health and reduce the numbers of people with multimorbidity in the future. Some additional ways that may counteract the difficulties patients with multiple chronic conditions face include embedding public health and health promotion in all aspects of society and having longer and more comprehensive primary care visits, especially for older adults [49].

### Strengths and limitations

Twitter appears to be a useful platform by which to engage nurses on important topics facing the profession and gain their feedback in an efficient and effective way. Several senior nurses, many of whom were experienced clinicians, educators and researchers, took part in the Twitter chat and their informed views provided insights into nursing in an age of multimorbidity, helping improve the robustness of the results. The limitations of this approach include the small sample size, comprising only of nurses many of whom were managers or academics, and participants who self-selected to take part in the discussion on social media. This included several FNF chairs, who comprised 12.5% of the sample, which may limit the generalisability of results. In addition, the short time period of the online chat and the potential unrepresentativeness of participants due to age linked variability of the use of Twitter, which is more prevalent in younger populations, may have introduced bias in the findings. This could further reduce the generalisability of results.

## Conclusion

Social media platforms such as Twitter might be a useful way to engage healthcare professionals in discussing important issues related to practice. The findings of the online chat suggest that multimorbidity is a growing problem which nurses can help address. Investing in research to understand how multiple chronic conditions develop and impact people lives may improve nursing education and clinical practice. Better assessment tools and clinical guidelines that focus on comorbidities could be developed to guide nurses in delivering holistic, person-centred care.

## Abbreviations

CAF: Common Assessment Framework
COPD: Chronic Obstructive Pulmonary Disease
CPD: Continuing Professional Development
CYP: Children and Young People
FNF: Florence Nightingale Foundation
LTCs: Long-term Conditions
MDTs: Multi-Disciplinary Teams
MUS: Medically Unexplained Symptoms
NHS: National Health Service
NICE: National Institute for Health and Care Excellence
UK: United Kingdom
